# A phase III randomized study to evaluate the efficacy and safety of CT-P13 compared with reference infliximab in patients with active rheumatoid arthritis: 54-week results from the PLANETRA study

**DOI:** 10.1186/s13075-016-0981-6

**Published:** 2016-04-02

**Authors:** Dae Hyun Yoo, Artur Racewicz, Jan Brzezicki, Roman Yatsyshyn, Edgardo Tobias Arteaga, Asta Baranauskaite, Carlos Abud-Mendoza, Sandra Navarra, Vladimir Kadinov, Irmgadt Goecke Sariego, Seung Suh Hong, Sung Young Lee, Won Park

**Affiliations:** Hanyang University Hospital, Seoul, Republic of Korea; NZOZ Osteo-Medic, Bialystok, Poland; Wojewodzki Szpital Zespolony, Elblag, Poland; Ivano-Frankivsk Regional Clinical Hospital, Ivano-Frankivsk, Ukraine; Hospital Militar Central, Bogota, Colombia; Lithuanian University of Health Sciences, Kaunas, Lithuania; Hospital Central Dr. Ignacio Morones Prieto, San Luis Potosi, Mexico; St. Luke’s Medical Center, Quezon City, Philippines; University Hospital St. Marina, Varna, Bulgaria; Prosalud y Cia Ltda, Santiago, Chile; CELLTRION, Incheon, Republic of Korea; Inha University Hospital, Incheon, Republic of Korea

**Keywords:** Biosimilar, CT-P13, Infliximab, Rheumatoid arthritis, Efficacy, Immunogenicity, Safety, Pharmacokinetics, ACR20, Sharp score

## Abstract

**Background:**

CT-P13 (Remsima®, Inflectra®) is a biosimilar of the infliximab reference product (RP; Remicade®). The aim of this study was to compare the 54-week efficacy, immunogenicity, safety, pharmacokinetics (PK) and pharmacodynamics (PD) of CT-P13 and RP in patients with active rheumatoid arthritis (RA).

**Methods:**

In this multinational phase III double-blind study, patients with active RA and an inadequate response to methotrexate (MTX) were randomized (1:1) to receive CT-P13 (3 mg/kg) or RP (3 mg/kg) at weeks 0, 2, 6 and then every 8 weeks to week 54 in combination with MTX (12.5–25 mg/week). Efficacy endpoints included American College of Rheumatology (ACR)20, ACR50 and ACR70 response rates, Disease Activity Score in 28 joints (DAS28), Simplified Disease Activity Index (SDAI), Clinical Disease Activity Index (CDAI), European League Against Rheumatism (EULAR) response rates, patient-reported outcomes and joint damage progression. Immunogenicity, safety and PK/PD outcomes were also assessed.

**Results:**

Of 606 randomized patients, 455 (CT-P13 233, RP 222) were treated up to week 54. At week 54, ACR20 response rate was highly similar between groups (CT-P13 74.7 %, RP 71.3 %). ACR50 and ACR70 response rates were also comparable between groups (CT-P13 43.6 % and 21.3 %, respectively; RP 43.1 % and 19.9 %, respectively). DAS28, SDAI and CDAI decreased from baseline to week 54 to a similar extent with CT-P13 and RP. Radiographic progression measured by Sharp scores as modified by van der Heijde was also comparable. With both treatments, patient assessments of pain, disease activity and physical ability, as well as mean scores on the Medical Outcomes Study Short Form Health Survey (SF-36), improved markedly at week 14 and remained stable thereafter up to week 54. The proportion of patients positive for antidrug antibodies at week 54 was similar between the two groups: 41.1 % and 36.0 % with CT-P13 and RP, respectively. CT-P13 was well tolerated and had a similar safety profile to RP. PK/PD results were also comparable between CT-P13 and RP.

**Conclusions:**

CT-P13 and RP were comparable in terms of efficacy (including radiographic progression), immunogenicity and PK/PD up to week 54. The safety profile of CT-P13 was also similar to that of RP.

**Trial registration:**

ClinicalTrials.gov identifier: NCT01217086. Registered 4 Oct 2010.

**Electronic supplementary material:**

The online version of this article (doi:10.1186/s13075-016-0981-6) contains supplementary material, which is available to authorized users.

## Background

Infliximab is a chimeric, anti-tumor necrosis factor (TNF) monoclonal antibody proven to be effective in patients with active rheumatoid arthritis (RA) not responding to methotrexate (MTX) [1, 2]. Over the past 15 years, the introduction of infliximab and other biologics has led to dramatic improvements in the management of RA, particularly in terms of enabling patients to achieve better outcomes [3]. However, there is an imbalance in the availability and affordability of these biologics worldwide due to their high costs [4, 5].

Some biologics, including infliximab, are at the end of their patent period. This fact, together with the high costs of these drugs, has prompted the recent development of biosimilar drugs. Biosimilars are highly similar to already approved innovator or “reference” biologics in terms of structure, efficacy, safety and quality [6, 7]. By virtue of their lower price, biosimilars have the potential to reduce healthcare costs relative to reference biologics [8–11], thereby increasing access to biologic drugs for patients who require them.

CT-P13 (Remsima®, Inflectra®) is a biosimilar of reference infliximab (Remicade®), hereafter referred to as the *reference product* (RP). It has been approved by the European Medicines Agency for use in all the indications for which RP is licensed, including RA. CT-P13 and RP are the same in terms of their pharmaceutical form, strength, composition and route of administration [12]. Consequently, dosage and administration instructions for CT-P13 are the same as those for RP. Nonclinical evaluations have shown that CT-P13 and RP are comparable with regard to the potency of TNF neutralization, the key mechanism of action of infliximab, in WEHI 164 cells [12]. A number of other in vitro assays have also demonstrated the similarity of CT-P13 and RP in terms of levels of apoptosis and complement-dependent cytotoxicity in a transmembrane TNF-expressing Jurkat cell line, and of antibody-dependent cellular cytotoxicity using peripheral blood mononuclear cells or whole blood from patients with Crohn’s disease [12, 13].

The PLANETRA (*P*rogramme eva*L*uating the *A*utoimmune disease i*N*v*E*stigational drug c*T*-p13 in *RA* patients) study was performed to assess the equivalence in efficacy of CT-P13 and RP treatment in patients with active RA. The primary 30-week findings proved equivalency in efficacy outcomes between CT-P13 and RP in terms of American College of Rheumatology (ACR) response (intent-to-treat population) [14]. Safety, pharmacokinetics (PK) and pharmacodynamics (PD) profiles were also comparable between the two drugs. As reported here, the PLANETRA study researchers also evaluated the extended effects of CT-P13 compared with RP in patients with active RA up to 54 weeks, including efficacy, radiographic progression, immunogenicity, safety, PK and PD.

## Methods

Full details of the methods of this study have been reported previously [14].

### Patients

Patients aged 18–75 years were included if they had been diagnosed with RA according to the revised 1987 ACR classification criteria for ≥1 year before screening. Active disease was defined as having at least six swollen joints, at least six tender joints and at least two of the following: morning stiffness for at least 45 minutes, erythrocyte sedimentation rate (ESR) >28 mm/h or serum C-reactive protein (CRP) concentration >2.0 mg/dl. Eligible patients had not responded adequately to MTX for ≥3 months and were required to have received a stable MTX dose (12.5–25 mg/week) for ≥4 weeks before screening.

### Study design and treatment

This randomized, double-blind, multicenter, multinational, parallel-group, prospective phase III study (ClinicalTrials.gov identifier NCT01217086) was performed at 100 study centers across 19 countries in Europe, Asia, Latin America and the Middle East. Patients were randomly assigned (1:1 ratio) to receive 3 mg/kg of CT-P13 (CELLTRION Inc, Incheon, Republic of Korea) or RP (Janssen Biotech Inc, Horsham, PA, USA) via a 2-h intravenous infusion. Patients were treated at weeks 0, 2 and 6 and then received a further six infusions every 8 weeks until week 54. Patients were premedicated with an antihistamine as needed. MTX and folic acid were coadministered to all patients. The study was conducted according to the Declaration of Helsinki and International Committee on Harmonisation Good Clinical Practice guidelines. The protocol was approved by regulatory authorities and the ethics committees of each study site. All patients provided written informed consent.

### Study endpoints and assessments

Efficacy, PK, PD and safety parameters were evaluated up to week 54. The primary endpoint of this study was the ACR20 response rate at week 30. Equivalence of efficacy according to ACR20 criteria was concluded if the 95 % confidence interval (CI) for the treatment difference was within ±15 %. Efficacy parameters were evaluated at baseline and at weeks 14, 30 and 54. Eligible patients were followed for ACR20, ACR50, ACR70, Disease Activity Score in 28 joints (DAS28), Simplified Disease Activity Index (SDAI), Clinical Disease Activity Index (CDAI) and European League Against Rheumatism (EULAR) response rate. Patient-reported outcomes (PROs) were evaluated using patient assessment of pain on the visual analogue scale (VAS), VAS patient global assessment of disease activity, the Health Assessment Questionnaire (HAQ) estimate of physical ability and the Medical Outcomes Study Short Form Health Survey (SF-36). To assess joint damage progression (JDP), radiographs obtained at baseline and at week 54 were evaluated with the “paired review” method; this evaluation was performed by two independent readers without knowledge of the time point of the radiographs. Individual component scores for JDP were calculated according to the van der Heijde modification of the Sharp scoring system, and used the mean score of two readers [15]. With respect to immunogenicity, the proportion of patients positive for antidrug antibodies (ADAs) was evaluated at screening and at weeks 14, 30 and 54 [14]. The neutralizing activity of ADAs (NAbs) was also assessed by a flow-through immunoassay method using the Gyros immunoassay platform (Gyros AB, Uppsala, Sweden). For safety, adverse events (AEs) and changes in laboratory parameters were monitored throughout the study. A treatment-emergent AE (TEAE) was defined as any event not present before exposure to study treatment or an event that worsened in intensity or frequency after exposure to study treatment. At each planned visit, patients were screened for tuberculosis (TB) by clinical examination with careful history taking. For patients living in countries with an increased TB prevalence, interferon γ-release assay (IGRA; QuantiFERON-TB Gold-IT, QIAGEN, Hilden, Germany) was rechecked at weeks 14, 30 and 54 to identify positive conversion from negative results at baseline [14]. Latent TB was defined as a positive conversion of IGRA together with a negative examination on chest x-ray. Patients were monitored for infusion-related reactions, including hypersensitivity and anaphylactic reaction.

PK evaluations included mean maximum serum drug concentration and mean minimum serum drug concentration. PD evaluations included CRP and ESR. Serum blood samples were obtained immediately before dosing, at the end of the infusion and 1 h after the infusion for PK assessments, and immediately before dosing for PD assessments.

Post hoc efficacy endpoints included the proportion of patients achieving ACR/EULAR remission by visit (Boolean-based, defined as swollen joint count [SJC] and tender joint count [TJC] less than or equal to one, CRP ≤1 mg/dl, and patient global VAS ≤1 using a 0–10 scale; or index-based, defined as SDAI ≤3.3) and the proportion of patients who had no radiographic progression in total Sharp score (≤0 or ≤0.5 units of change from baseline).

### Statistical analyses

Statistical analyses were performed using SAS software version 9.1.3 or later (SAS Institute, Cary, NC, USA). Efficacy analyses were performed in the per-protocol (PP) population, which included all randomly assigned patients who did not have any major protocol deviations, using the as-observed method. The JDP analysis was performed in the intent-to-treat population (ITT), which included all randomly assigned patients. ACR response rates were also evaluated in the ITT population, as well as the PP population, using nonresponder imputation (NRI) for missing values. Exact binomial analyses and sensitivity analyses were performed for the ACR endpoints. Descriptive statistics were performed for DAS28, SDAI, CDAI, EULAR response, JDP, PROs including patient assessment of pain using the VAS, patient assessment of disease activity using the VAS, HAQ and SF-36. EULAR response criteria were analyzed using a proportional odds model stratified by region and CRP. Analysis of DAS28 was by analysis of covariance (ANCOVA) with treatment as a fixed effect and baseline DAS28, region and CRP category as covariates. Additional analyses were carried out for assessment of JDP, including patients with missing data (incomplete images) at week 54. Details of the methods have been reported previously [16].

Safety assessments were conducted in all patients who received at least one part infusion of study drug (safety population). The PK-PD population consisted of all patients who received either study drug during the 30-week blinded study period and had at least one PK-PD concentration data value.

Sensitivity analyses included ACR responses using a last observation carried forward (LOCF) approach in the ITT population; descriptive statistics of DAS28, SDAI, CDAI and EULAR response; and PROs in the ITT population. For post hoc efficacy endpoints, NRI was used for the calculation of ACR/EULAR remission rates. Linear interpolation or extrapolation was used to calculate the proportion of patients who had no radiographic progression in total Sharp score.

## Results

### Patient disposition and baseline characteristics

**Fig. 1:**
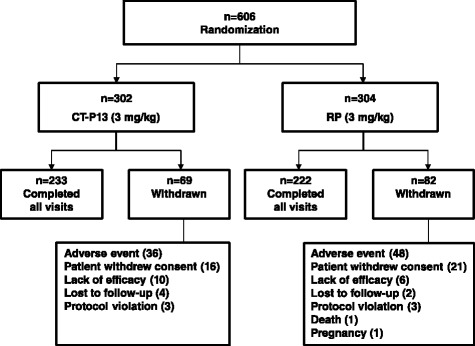
Patient disposition. A total of 606 patients were randomized into either the CT-P13 group (*n* = 302) or the RP group (*n* = 304). A total of 151 patients were withdrawn for the stated reasons. The first patient was screened in November 2010, and the last patient visit was in July 2012. *RP* reference product (i.e., reference infliximab)

Of the 606 randomized patients, most (*n* = 455) were treated up to week 54: 233 (77.2 %) of 302 with CT-P13 and 222 (73.0 %) of 304 with RP (Fig. 1). As has been reported previously [14], baseline patient demographics and disease characteristics were similar between the two groups (Table 1).

**Table 1:** Baseline patient demographics and disease characteristics

	CT-P13 3 mg/kg (*n* = 302)	RP 3 mg/kg (*n* = 304)	Total (*N* = 606)
Age, median, years (range)	50 (18–75)	50 (21–74)	50 (18–75)
Sex, *n* (%)			
Female	245 (81.1)	256 (84.2)	501 (82.7)
Male	57 (18.9)	48 (15.8)	105 (17.3)
Ethnicity, *n* (%)			
Asian	34 (11.3)	37 (12.2)	71 (11.7)
Black	2 (0.7)	1 (0.3)	3 (0.5)
White	220 (72.8)	222 (73.0)	442 (72.9)
Other	46 (15.2)	44 (14.5)	90 (14.9)
Height, cm, median (range)	162.3 (144.0–186.0)	162.0 (124.0–190.0)	162.0 (124.0–190.0)
Weight, kg, median (range)	69.0 (36.5–134.0)	68.0 (36.0–136.0)	68.6 (36.0–136.0)
BMI, kg/m^2^, median (range)	26.3 (13.9–49.8)	25.4 (15.0–53.1)	25.9 (13.9–53.1)
Anti-CCP antibody-positive, *n* (%)	228 (75.5)	233 (76.6)	461 (76.1)
IgA RF-positive, *n* (%)	131 (43.4)	134 (44.1)	265 (43.7)
IgM RF-positive, *n* (%)	225 (74.5)	220 (72.4)	445 (73.4)
IgG RF-positive, *n* (%)	173 (57.3)	171 (56.3)	344 (56.8)
Joint count			
TJC, 68 joints	25.6 ± 13.9	24.0 ± 12.9	24.8 ± 13.4
SJC, 66 joints	16.2 ± 8.7	15.2 ± 8.3	15.7 ± 8.5
TJC, 28 joints	15.9 ± 6.4	15.1 ± 6.1	15.5 ± 6.2
SJC, 28 joints	12.0 ± 4.9	11.2 ± 4.7	11.6 ± 4.8
Duration of prior MTX therapy, weeks	97.7 ± 141.2	89.4 ± 96.5	93.6 ± 120.8
MTX dose, mg	15.6 ± 3.1	15.6 ± 3.2	15.6 ± 3.1
CDAI	40.9 ± 11.5	39.3 ± 11.1	40.1 ± 11.3
SDAI	42.8 ± 11.9	41.2 ± 11.7	42.0 ± 11.8
CRP, mg/dl	1.9 ± 2.5	1.9 ± 2.2	1.9 ± 2.4
ESR, mm/h	46.6 ± 22.4	48.5 ± 22.6	47.5 ± 22.5
DAS28-CRP	5.9 ± 0.8	5.8 ± 0.9	5.8 ± 0.9
HAQ	1.6 ± 0.6	1.6 ± 0.6	1.6 ± 0.6
Patient assessment of pain	65.9 ± 17.4	65.5 ± 17.2	65.7 ± 17.3
Patient global assessment of disease activity	65.7 ± 17.2	65.4 ± 17.0	65.5 ± 17.1
Physician global assessment of disease activity	64.7 ± 14.3	65.0 ± 13.5	64.8 ± 13.9

*BMI* body mass index, *CCP* cyclic citrullinated peptide, *CDAI* Clinical Disease Activity Index, *CRP* C-reactive protein, *DAS28* Disease Activity Score in 28 joints, *ESR* erythrocyte sedimentation rate, *HAQ* Health Assessment Questionnaire, *Ig* immunoglobulin, *MTX* methotrexate, *RF* rheumatoid factor, *RP* reference product (i.e., reference infliximab), *SD* standard deviation, *SDAI* Simplified Disease Activity Index, *SJC* swollen joint count, *TJC* tender joint count

Except where indicated otherwise, values are mean ± SD

### Efficacy

**Fig. 2:**
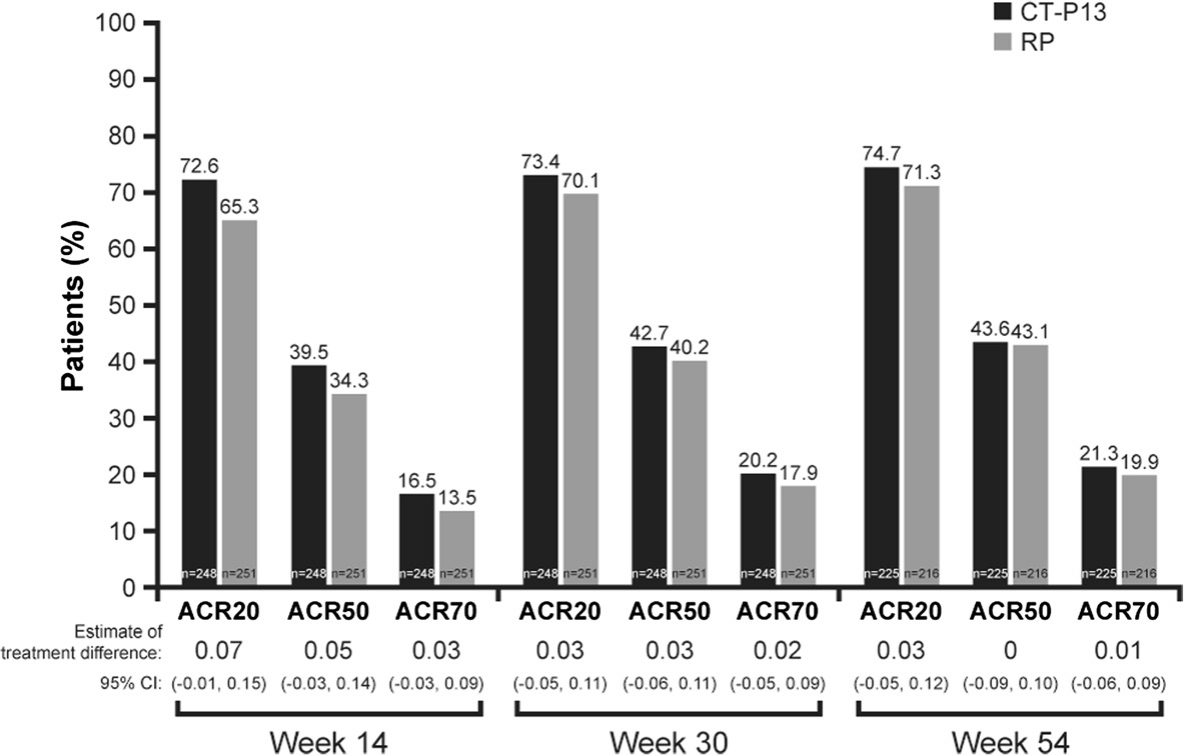
American College of Rheumatology (ACR) response rates over time in the per-protocol population, with nonresponder imputation approach. To estimate the difference in proportions between the two treatment groups, we used the exact binomial test. ACR20, ACR50 and ACR70 denote the ACR 20 %, 50 % and 70 % improvement criteria, respectively. *CI* confidence interval, *RP* reference product (i.e., reference infliximab)

Overall, efficacy results for CT-P13 up to week 54 were generally comparable to those observed with RP. No statistical differences were observed for any efficacy endpoint. The primary endpoint of PLANETRA (ACR20 response at week 30) was equivalent between the two treatment groups [14]. The ACR20, ACR50 and ACR70 treatment responses were analogous between the two treatment groups throughout the course of the study (Fig. 2). At week 54 in the CT-P13 versus RP groups, the ACR20, ACR50 and ACR70 responses in the PP population were 74.7 % (168 of 225) versus 71.3 % (154 of 216), 43.6 % (98 of 225) versus 43.1 % (93 of 216) and 21.3 % (48 of 225) versus 19.9 % (43 of 216), respectively. ACR responses in the ITT population are shown in Additional file 1 and were also similar between the two treatment groups, regardless of the method of missing data imputation (NRI or LOCF).

**Fig. 3:**
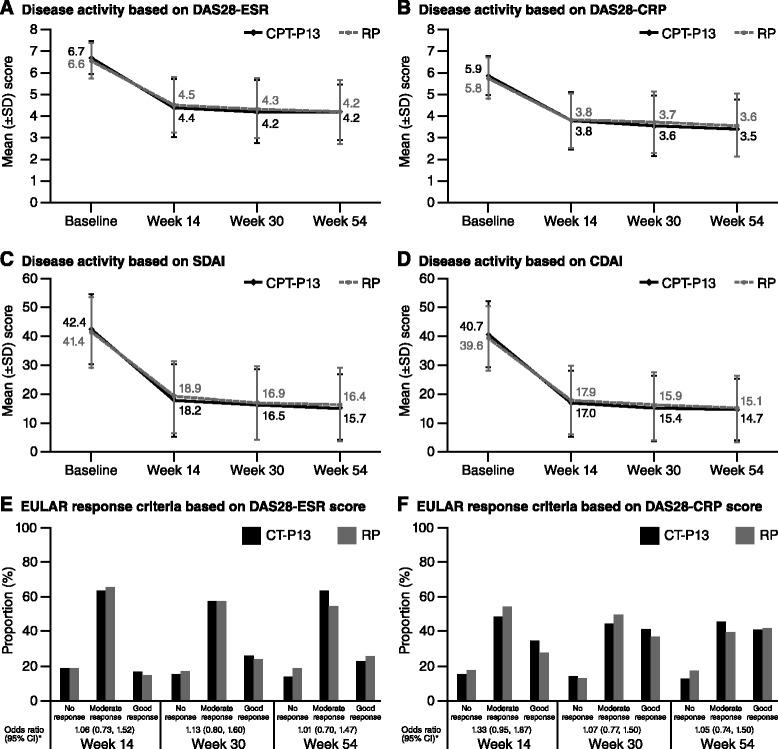
Changes in efficacy parameters over time with CT-P13 and RP in the per-protocol population. **a** Disease activity based on DAS28-ESR. **b** Disease activity based on DAS28-CRP. **c** Disease activity based on SDAI. **d** Disease activity based on CDAI. **e** EULAR response criteria based on DAS28-ESR score. **f** EULAR response criteria based on DAS28-CRP score. *Proportional odds model with EULAR as response, treatment as a fixed effect, and region and CRP category as covariates. An odds ratio >1 implied that a patient who received CT-P13 had a higher likelihood of EULAR response than a patient who received RP. The proportional odds assumption implied that the relationship between each pair of outcome responses was the same. *CDAI* Clinical Disease Activity Index, *CI* confidence interval, *CRP* C-reactive protein, *DAS28* Disease Activity Score in 28 joints, *ESR* erythrocyte sedimentation rate, *EULAR* European League Against Rheumatism, *RP* reference product (i.e., reference infliximab), *SD* standard deviation, *SDAI* Simplified Disease Activity Index

Disease activity as measured by DAS28-ESR, DAS28-CRP, SDAI and CDAI decreased from baseline through to week 54 in the PP population as well as in the ITT population (Fig. 3a–d and Additional file 2A–D). For all of these parameters, the reductions were comparable between the CT-P13 and RP treatment groups. In the CT-P13 group at week 54, the mean decreases from baseline in DAS28-ESR, DAS28-CRP, SDAI and CDAI scores were 2.4, 2.3, 26.3 and 25.7, respectively, in the PP population. The respective mean values for RP were 2.4, 2.2, 24.6 and 24.0. There was no evidence of a difference between the two treatment groups in change from baseline in DAS28-ESR or DAS28-CRP at each time point at the 5 % level of significance (DAS28-ESR treatment differences [95 % CIs] −0.11 [−0.31, 0.10] at week 14, −0.10 [−0.33, 0.13] at week 30 and −0.05 [−0.30, 0.19] at week 54; DAS28-CRP treatment differences −0.15 [−0.34, 0.05] at week 14, −0.06 [−0.27, 0.16] at week 30 and −0.07 [−0.30, 0.17] at week 54 in the PP population). Likewise, the proportion of patients with a good and moderate EULAR response to treatment was similar between the two treatment groups throughout the study in both the PP and the ITT populations (EULAR [ESR] and EULAR [CRP]) (Fig. 3e–f and Additional file 2E–F). With CT-P13 at week 54, 86.0 % (191 of 222) and 87.4 % (194 of 222) of patients had a moderate or good response according to EULAR (ESR) and EULAR (CRP) criteria, respectively, in the PP population. Respective values for RP were 81.2 % (177 of 218) and 82.5 % (179 of 217), respectively, in the PP population.

In terms of PROs (Table 2 and Additional file 3), mean decreases in VAS score for patient assessment of pain from baseline were similar between the two treatment groups in both the PP population and the ITT population. Mean baseline VAS scores for patient assessment of pain for CT-P13 and RP, respectively, were 65.7 and 65.5 in the PP population. Respective mean decreases from baseline were 29.6 versus 27.1 at week 14, 29.5 versus 27.7 at week 30 and 30.2 versus 28.4 at week 54. For patient global assessment of disease activity, mean decreases from baseline were also similar in both groups. The respective mean baseline VAS scores for patient global assessment of disease activity for CT-P13 and RP were 65.1 versus 65.3 in the PP population. Respective mean decreases from baseline were 29.5 versus 25.5 at week 14, 28.1 versus 26.9 at week 30 and 30.3 versus 26.6 at week 54. Mean score for the HAQ estimate of physical ability decreased from baseline at weeks 14, 30 and 54 in each treatment arm. Mean decreases from baseline were again similar in both groups. The respective mean baseline HAQ estimate of physical ability for CT-P13 and RP was 1.61 versus 1.54 in the PP population. The respective mean decreases from baseline were 0.59 versus 0.50 at week 14, 0.60 versus 0.51 at week 30 and 0.60 versus 0.52 at week 54.

**Table 2:** Improvement in patient-reported outcomes with CT-P13 and RP in the per-protocol population

	CT-P13 (3 mg/kg)			RP (3 mg/kg)		
Time point	*n*	Actual result (mean ± SD)	Change from baseline (mean ± SD)	*n*	Actual result (mean ± SD)	Change from baseline (mean ± SD)
VAS score for the patient assessment of pain						
Baseline	248	65.7 ± 17.8	–	251	65.5 ± 17.7	–
Week 14	248	36.1 ± 21.4	−29.6 ± 23.3	250	38.3 ± 22.2	−27.1 ± 23.1
Week 30	248	36.2 ± 22.9	−29.5 ± 25.6	250	37.7 ± 23.6	−27.7 ± 24.9
Week 54	226	35.0 ± 21.2	−30.2 ± 23.8	220	37.4 ± 24.7	−28.4 ± 26.9
VAS score for the patient global assessment of disease activity						
Baseline	248	65.1 ± 17.5	–	251	65.3 ± 17.3	–
Week 14	248	35.6 ± 21.1	−29.5 ± 22.1	249	39.7 ± 22.5	−25.5 ± 24.4
Week 30	247	37.0 ± 22.3	−28.1 ± 25.9	250	38.4 ± 23.4	−26.9 ± 25.5
Week 54	225	34.9 ± 20.7	−30.3 ± 24.3	220	38.7 ± 25.3	−26.6 ± 27.8
HAQ estimate of physical ability						
Baseline	248	1.61 ± 0.56	–	251	1.54 ± 0.58	–
Week 14	248	1.02 ± 0.62	−0.59 ± 0.55	251	1.04 ± 0.64	−0.50 ± 0.50
Week 30	248	1.01 ± 0.64	−0.60 ± 0.60	251	1.03 ± 0.66	−0.51 ± 0.56
Week 54	226	0.99 ± 0.61	−0.60 ± 0.61	220	1.02 ± 0.64	−0.52 ± 0.59
SF-36 score (physical component summary)						
Baseline	247	31.4 ± 6.1	–	251	31.8 ± 7.2	–
Week 14	247	38.9 ± 7.6	7.5 ± 7.1	251	37.6 ± 7.9	5.8 ± 6.8
Week 30	248	38.6 ± 7.9	7.1 ± 7.9	250	38.3 ± 8.0	6.5 ± 7.6
Week 54	226	39.2 ± 7.5	7.6 ± 8.1	220	38.6 ± 8.7	6.6 ± 8.4
SF-36 score (mental component summary)						
Baseline	247	36.8 ± 10.4	–	251	38.4 ± 11.3	–
Week 14	247	43.4 ± 10.7	6.6 ± 10.2	251	44.9 ± 9.6	6.5 ± 10.4
Week 30	248	44.0 ± 10.2	7.1 ± 10.0	251	45.0 ± 10.3	6.6 ± 10.4
Week 54	226	43.9 ± 9.9	7.1 ± 10.1	220	45.1 ± 10.0	6.9 ± 11.2

*HAQ* Health Assessment Questionnaire, *RP* reference product (i.e., reference infliximab), *SD* standard deviation, *SF-36* Medical Outcomes Study Short Form Health Survey, *VAS* visual analogue scale (mm)

**Fig. 4:**
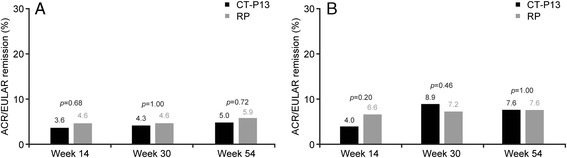
Proportion of patients with ACR/EULAR remission in the intent-to-treat population, with nonresponder imputation approach. **a** ACR/EULAR remission by Boolean-based criterion. **b** ACR/EULAR remission by index-based criterion (SDAI). *p* value was calculated using Fisher’s exact test. *ACR* American College of Rheumatology, *EULAR* European League Against Rheumatism, *RP* reference product (i.e., reference infliximab), *SDAI* Simplified Disease Activity Index

Throughout the study, mean SF-36 score increased from baseline to week 54 in both groups. Scores were similar between CT-P13 and RP groups for all components of the SF-36. For the physical component summary, the mean increase from baseline to week 54 was 7.6 and 6.6 with CT-P13 and RP, respectively, in the PP population. For the mental component summary, the mean increase from baseline to week 54 was 7.1 and 6.9 with CT-P13 and RP, respectively, in the PP population. The proportion of patients achieving remission was similar between groups throughout the study in both Boolean-based and index-based (SDAI) ACR/EULAR remission criteria in the ITT population using the NRI approach (Fig. 4).

### Radiologic progression

In total, 336 patients had radiographs at both baseline and week 54 and were eligible for assessment of JDP. Mean total Sharp scores at baseline were 68.3 in the CT-P13 group and 64.8 in the RP group. The mean change from baseline to week 54 in JDP was similar between the CT-P13 and RP treatment groups with respect to total Sharp score (1.0 ± 6.3 versus 0.6 ± 5.6, *p* = 0.5463), joint space narrowing score (0.4 ± 4.2 versus 0.7 ± 4.0, *p* = 0.4852) and erosion score (0.7 ± 3.9 versus 0.0 ± 3.4, *p* = 0.0795). Changes in total Sharp score from baseline to week 54 were 1.3 ± 9.3 and 0.7 ± 7.0 for CT-P13 and RP (*p* = 0.3171), respectively, when ANCOVA was used. There was no impact of the choice of imputation (ANCOVA, simple linear extrapolation, or no missing data imputation).

The proportion of patients who had no radiographic progression in total Sharp score was similar in both groups (≤0 units of change from baseline: 51.7 % [153 of 296] and 51.4 % [151 of 294] in the CT-P13 and RP groups, respectively [*p* = 1.0000]; ≤0.5 units of change from baseline: 58.1 % [172 of 296] and 57.8 % [170 of 294] [*p* = 1.0000], respectively, with linear interpolation or extrapolation).

### Immunogenicity

The proportion of patients positive for ADAs at week 54 was similar between the two treatment groups: 124 (41.1 %) and 108 (36.0 %) in the CT-P13 and RP groups, respectively. In general, almost all patients with a positive ADA result were also positive for NAbs.

### Safety

Among 606 patients randomized (302 and 304 patients in the CT-P13 and RP groups, respectively), 300 and 302 patients initiated study treatment. Owing to incorrect kits’ being dispensed, two patients in the RP group received one dose of CT-P13. Therefore, the safety population comprised 302 patients in the CT-P13 group and 300 in the RP group.

In general, CT-P13 was well tolerated and had a safety profile similar to that of RP. Overall, TEAEs were reported for 213 patients (70.5 %) in the CT-P13 group and 211 patients (70.3 %) in the RP group. TEAEs considered to be related to treatment occurred in 132 patients (43.7 %) in the CT-P13 group and 135 patients (45.0 %) in the RP group. The most frequently reported drug-related TEAEs (Table 3), occurring in at least nine patients in either treatment arm, were as follows in order of frequency: (1) for CT-P13, infusion-related reaction, upper respiratory tract infection, latent TB, abnormal liver function test, lower respiratory tract infection and urinary tract infection; and (2) for RP, infusion-related reaction, latent TB, upper respiratory tract infection, abnormal liver function test, urinary tract infection and lower respiratory tract infection. In the CT-P13 and RP treatment groups, infusion-related reactions were reported for 30 patients (9.9 %) and 43 patients (14.3 %), respectively.

**Table 3:** Treatment-related adverse events reported in at least 1 % of total patients

	CT-P13 (*n* = 302)	RP (*n* = 300)	Total CT-P13 + RP (*n* = 602)
Infusion-related reaction	30 (9.9)	43 (14.3)	73 (12.1)
Latent TB	22 (7.3)	20 (6.7)	42 (7.0)
Upper respiratory tract infection	23 (7.6)	14 (4.7)	37 (6.1)
Abnormal liver function test	22 (7.3)	14 (4.7)	36 (6.0)
Urinary tract infection	9 (3.0)	11 (3.7)	20 (3.3)
Lower respiratory tract infection	10 (3.3)	9 (3.0)	19 (3.2)
Flare in RA activity	7 (2.3)	5 (1.7)	12 (2.0)
Herpes virus infection	3 (1.0)	7 (2.3)	10 (1.7)
Anemia	4 (1.3)	5 (1.7)	9 (1.5)
Headache	4 (1.3)	5 (1.7)	9 (1.5)
Rash	2 (0.7)	4 (1.3)	6 (1.0)
Pyrexia	1 (0.3)	5 (1.7)	6 (1.0)

*RA* rheumatoid arthritis, *RP* reference product (i.e. reference infliximab), *TB* tuberculosis

Data are presented as count (percentage)

Active TB was reported in three patients (1 %) in the CT-P13 group and no patients in the RP group. Of the three patients with active TB, one patient was diagnosed with TB based on clinical judgment alone rather than on a positive identification of *Mycobacterium*. The proportion of patients who had a negative IGRA result at screening followed by a positive IGRA result during study drug exposure was similar (39 [12.9 %] and 38 [12.7 %] patients in the CT-P13 and RP groups, respectively). Among these, 28 (9.3 %) and 26 (8.7 %) patients, respectively, were reported as having a latent TB TEAE based on the judgment of investigators, and 24 (7.9 %) and 20 (6.7 %) patients, respectively, started TB prophylaxis with isoniazid.

The majority of TEAEs were mild to moderate in severity and did not lead to discontinuation. Treatment-emergent serious AEs were reported in 42 patients (13.9 %) in the CT-P13 group, and 31 patients (10.3 %) in the RP group. Among them, 23 patients (7.6 %) in the CT-P13 group and 14 patients (4.7 %) in the RP group were considered to have events related to treatment (Additional file 4). The proportion of patients who discontinued study treatment due to TEAEs was similar between the two groups: 33 patients (10.9 %) in the CT-P13 group and 47 patients (15.7 %) in the RP group; 29 (9.6 %) and 37 (12.3 %) patients, respectively, discontinued due to TEAEs considered to be related to treatment. Treatment-related events that caused discontinuation and occurred in more than one patient were infusion-related reaction (*n* = 14 [4.6 %]), TB (*n* = 3 [1.0 %]), anemia (*n* = 2 [0.7 %]), and abnormal liver function test (*n* = 2 [0.7 %]) in the CT-P13 group and infusion-related reaction (*n* = 17 [5.7 %]) and latent TB (*n* = 5 [1.7 %]) in the RP group. One death in the RP group was reported during the study. The cause was unknown and not considered to be related to the study treatment.

### Pharmacokinetics and pharmacodynamics

**Fig. 5:**
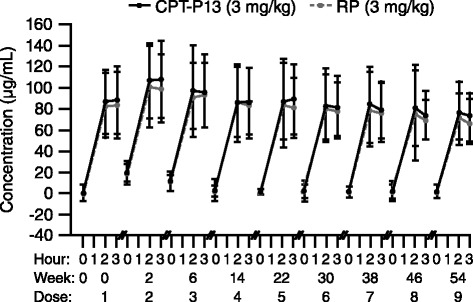
Serum concentrations of infliximab (mean ± SD) versus time in the CT-P13 and RP treatment groups in the pharmacokinetic population. *RP* reference product (i.e., reference infliximab), *SD* standard deviation

The PK and PD findings were highly comparable between the CT-P13 and RP groups. In the overall PK patient population, serum drug concentrations were similar throughout the study in the CT-P13 and RP groups (Fig. 5). In terms of PD, mean CRP and ESR levels decreased from baseline at all time points (weeks 14, 30 and 54). Respective mean decreases were similar in the CT-P13 and RP groups at week 54 for both CRP (0.67 ± 2.17 and 0.66 ± 2.66) and ESR (12.3 ± 22.13 and 15.2 ± 21.89).

## Discussion

The 54-week findings from this randomized, double-blind, multicenter, multinational, parallel-group, prospective phase III study confirm those previously reported at week 30 [14]. The results show that CT-P13 (3 mg/kg) and RP (3 mg/kg) are comparable in terms of efficacy, immunogenicity, safety and PK-PD in patients with active RA not responding adequately to MTX.

As previously reported for this study, the ACR20 response at week 30 (primary study endpoint) was equivalent for CT-P13 and RP, as the 95 % CIs for treatment difference were within the predefined margins for equivalence [14]. Similarly, at week 54, the efficacy of CT-P13 and RP was highly comparable across a broad range of measures of disease activity, including ACR responses and DAS28-ESR, DAS28-CRP, SDAI and CDAI, as well as PROs, including VAS scores for the patient assessment of pain and the patient global assessment of disease activity, HAQ scores and SF-36 scores.

Regarding the assessment of JDP, the handling of missing data and use of appropriate statistical techniques are important when analyzing radiographic results of a clinical trial. If several different methods of analysis give comparable results, the credibility of the data is increased [15]. CT-P13 showed comparable results when analyzed using either of two different missing data imputations or when missing data were excluded. The proportion of patients with no radiographic progression in total Sharp score was also similar in the two groups when using linear interpolation or extrapolation.

While there are limitations associated with comparing data across studies, the ACR responses we observed at week 54 were generally in the range of those reported in two previous randomized studies with RP in similar patient populations [16, 17]. For example, between weeks 52 and 54, ACR20 responses with RP ranged from 42 % to 56 % in these historical published studies (ITT analyses) [16, 17]. The ACR20 associated with CT-P13 in the present study was 57 % at week 54 (ITT analysis). In line with findings at week 0 [14], PK-PD parameters remained comparable between the CT-P13 and RP treatment groups at week 54. Serum drug concentrations and decreases in CRP and ESR levels were similar with both CT-P13 and RP throughout the study. These data are supported by the findings from the PLANETAS study [18], which also showed that CT-P13 and RP have similar effects on efficacy and PK-PD. The safety profile of CT-P13 observed in the present analysis was also similar to that of RP, and both agents were shown to be well tolerated through to week 54. The safety results were generally aligned with the findings from previous randomized trials of RP up to week 54 in patients with RA [16, 17, 19].

Data from the present study and a similar trial in patients with ankylosing spondylitis (PLANETAS) [18, 20] have shown that CT-P13 and RP possess comparable clinical efficacy, PK-PD and safety when used for over 1 year. Assuming that the biosimilar is priced less than the reference biologic—and in the European Union, biosimilars are generally approximately 30 % less expensive than reference products [21]—CT-P13 may prove to be a cost-effective alternative to infliximab RP. In this regard, a budget impact analysis recently assessed the potential effects of introducing CT-P13 for the treatment of RA in six central and eastern European countries. This analysis estimated savings of €20.8 million over 3 years if 80 % of patients taking RP switched to CT-P13, on the assumption that the price of CT-P13 is 75 % that of the RP [22]. In other countries, such as Norway and Denmark, biosimilar infliximab is recommended by tender systems and, indeed, it has rapidly increased market share in Norway due to the large difference in price between the reference product and the biosimilar [23]. The National Institute for Health and Care Excellence (NICE) in the United Kingdom changed its position on the use of infliximab for the treatment of ankylosing spondylitis by recommending the least expensive drug [24]. They also recommend using the least expensive infliximab for RA treatment [25]. The increasing use of biosimilars in the treatment of various inflammatory diseases may enhance reductions or discounts of the originator biologic’s price [21, 26]. This trend may finally increase accessibility to biologics and reduce total cost further.

The efficacy and safety of CT-P13 up to 102 weeks is being assessed in an extension of the present study, which is also evaluating switching treatment from RP to CT-P13 in patients with RA. The findings from the extension may support the longer-term efficacy and safety of CT-P13.

The main limitation of the present analysis is that the study sample size was calculated to analyze the primary endpoint at 30 weeks. Therefore, the prospective longer-term findings presented here at 54 weeks were not a key consideration when calculating the sample size.

## Conclusions

This randomized, multinational study in patients with active RA inadequately responding to MTX demonstrated that CT-P13 had comparable efficacy, immunogenicity and PK-PD to RP up to week 54. Additionally, CT-P13 was well tolerated, with a safety profile comparable to that of RP up to week 54.

## Additional files

Additional file 1: (A) American College of Rheumatology (ACR) response rates over time in the intent-to-treat population, with NRI approach. (B). American College of Rheumatology (ACR) response rates over time in the intent-to-treat population, with LOCF approach. To estimate the difference in proportions between the two treatment groups, we used the exact binomial test. ACR20, ACR50 and ACR70 denote the ACR 20 %, 50 % and 70 % improvement criteria, respectively. ACR, American College of Rheumatology; CI, confidence interval; LOCF, last observation carried forward; NRI, nonresponder imputation; RP, reference product (i.e. reference infliximab). (TIF 1024 kb) Additional file 2: Changes in efficacy parameters over time with CT-P13 and RP in the intent-to-treat population. (A) Disease activity based on DAS28-ESR; (B) Disease activity based on DAS28-CRP; (C) Disease activity based on SDAI; (D) Disease activity based on CDAI; (E) EULAR response criteria based on DAS28-ESR score; (F) EULAR response criteria based on DAS28-CRP score. *Proportional odds model with EULAR as response, treatment as a fixed effect, and region and CRP category as covariates. An odds ratio >1 implied that a patient who received CT-P13 had a higher likelihood of EULAR response than a patient who received RP. The proportional odds assumption implied that the relationship between each pair of outcome response was the same. CDAI, Clinical Disease Activity Index; CI, confidence interval; CRP, C-reactive protein; DAS28, Disease Activity Score in 28 joints; ESR, erythrocyte sedimentation rate; EULAR, European League Against Rheumatism; RP, reference product (i.e., reference infliximab); SD, standard deviation; SDAI, Simplified Disease Activity Index. (TIF 1420 kb) Additional file 3: Improvement in patient-reported outcomes with CT-P13 and RP in the intent-to-treat population. (DOC 52 kb) Additional file 4: Overview of treatment-related serious adverse events occurring by severity (n [%]). (DOC 49 kb)

## Abbreviations

ACR: American College of Rheumatology
ADA: antidrug antibody
AE: adverse event
ANCOVA: analysis of covariance
BMI: body mass index
CCP: cyclic citrullinated peptide
CDAI: Clinical Disease Activity Index
CI: confidence interval
CRP: C-reactive protein
DAS28: Disease Activity Score in 28 joints
ESR: erythrocyte sedimentation rate
EULAR: European League Against Rheumatism
HAQ: Health Assessment Questionnaire
Ig: immunoglobulin
IGRA: interferon γ-release assay
ITT: intent-to-treat
JDP: joint damage progression
LOCF: last observation carried forward
MTX: methotrexate
NAbs: neutralizing activity of antidrug antibodies
NICE: National Institute for Health and Care Excellence
NRI: nonresponder imputation
PD: pharmacodynamics
PK: pharmacokinetics
PLANETRA: *P*rogramme eva*L*uating the *A*utoimmune disease i*N*v*E*stigational drug c*T*-p13 in *RA* patients
PP: per-protocol
PRO: patient-reported outcome
RA: rheumatoid arthritis
RF: rheumatoid factor
RP: reference product
SD: standard deviation
SDAI: Simplified Disease Activity Index
SF-36: Medical Outcomes Study Short Form Health Survey
SJC: swollen joint count
TB: tuberculosis
TEAE: treatment-emergent adverse event
TJC: tender joint count
TNF: tumor necrosis factor
VAS: visual analogue scale
