# Supplementary figures and images for: A phase III randomized study to evaluate the efficacy and safety of CT-P13 compared with reference infliximab in patients with active rheumatoid arthritis: 54-week results from the PLANETRA study
Source: Arthritis Res Ther. 2016 Apr 2;18:82. doi: 10.1186/s13075-016-0981-6 (PMC4818886; doi:10.1186/s13075-016-0981-6)

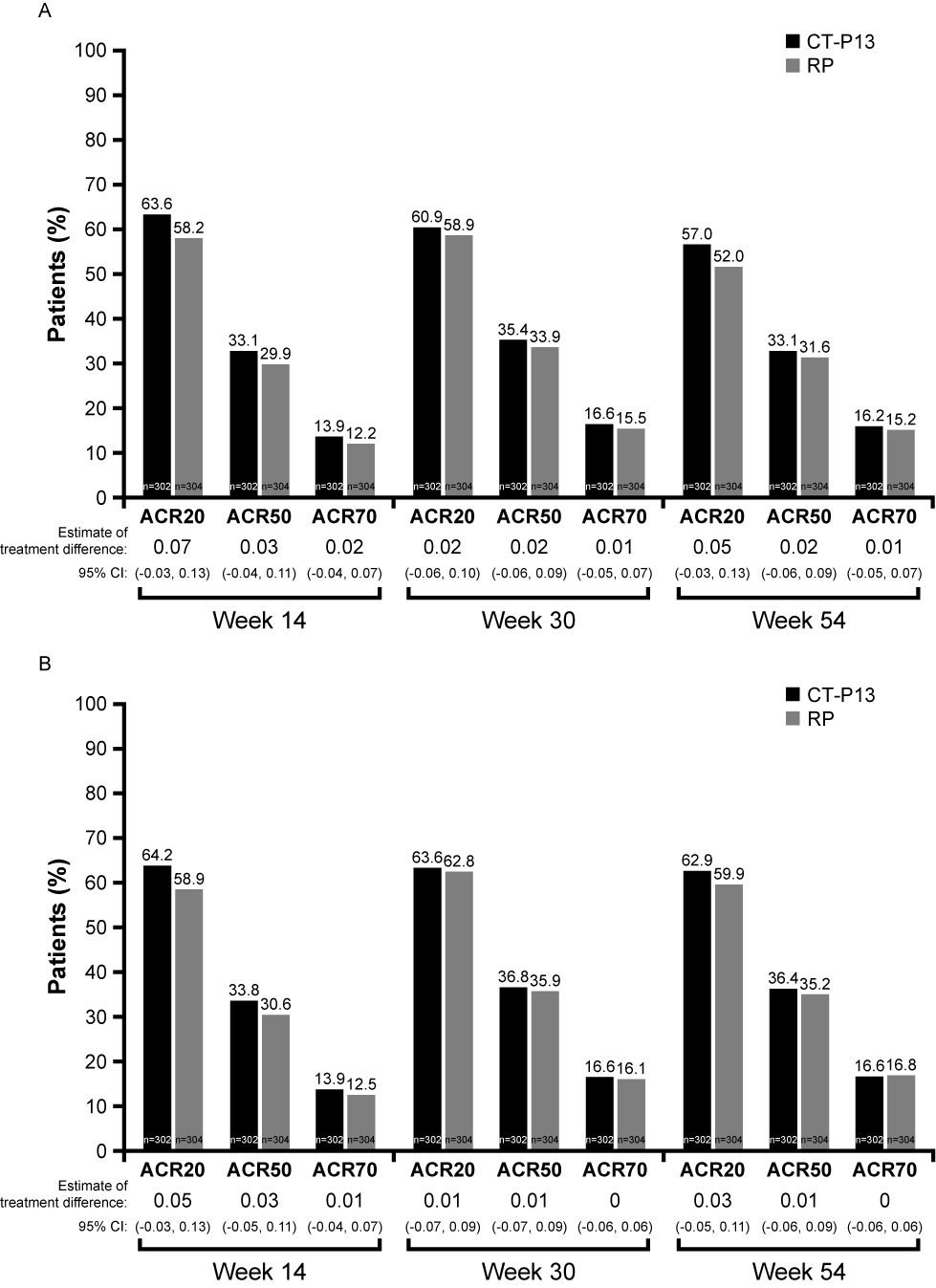

Supplement: Additional file 1: — (A) American College of Rheumatology (ACR) response rates over time in the intent-to-treat population, with NRI approach. (B). American College of Rheumatology (ACR) response rates over time in the intent-to-treat population, with LOCF approach. To estimate the difference in proportions between the two treatment groups, we used the exact binomial test. ACR20, ACR50 and ACR70 denote the ACR 20 %, 50 % and 70 % improvement criteria, respectively. ACR, American College of Rheumatology; CI, confidence interval; LOCF, last observation carried forward; NRI, nonresponder imputation; RP, reference product (i.e. reference infliximab). (TIF 1024 kb) [file 13075_2016_981_MOESM1_ESM.tif]

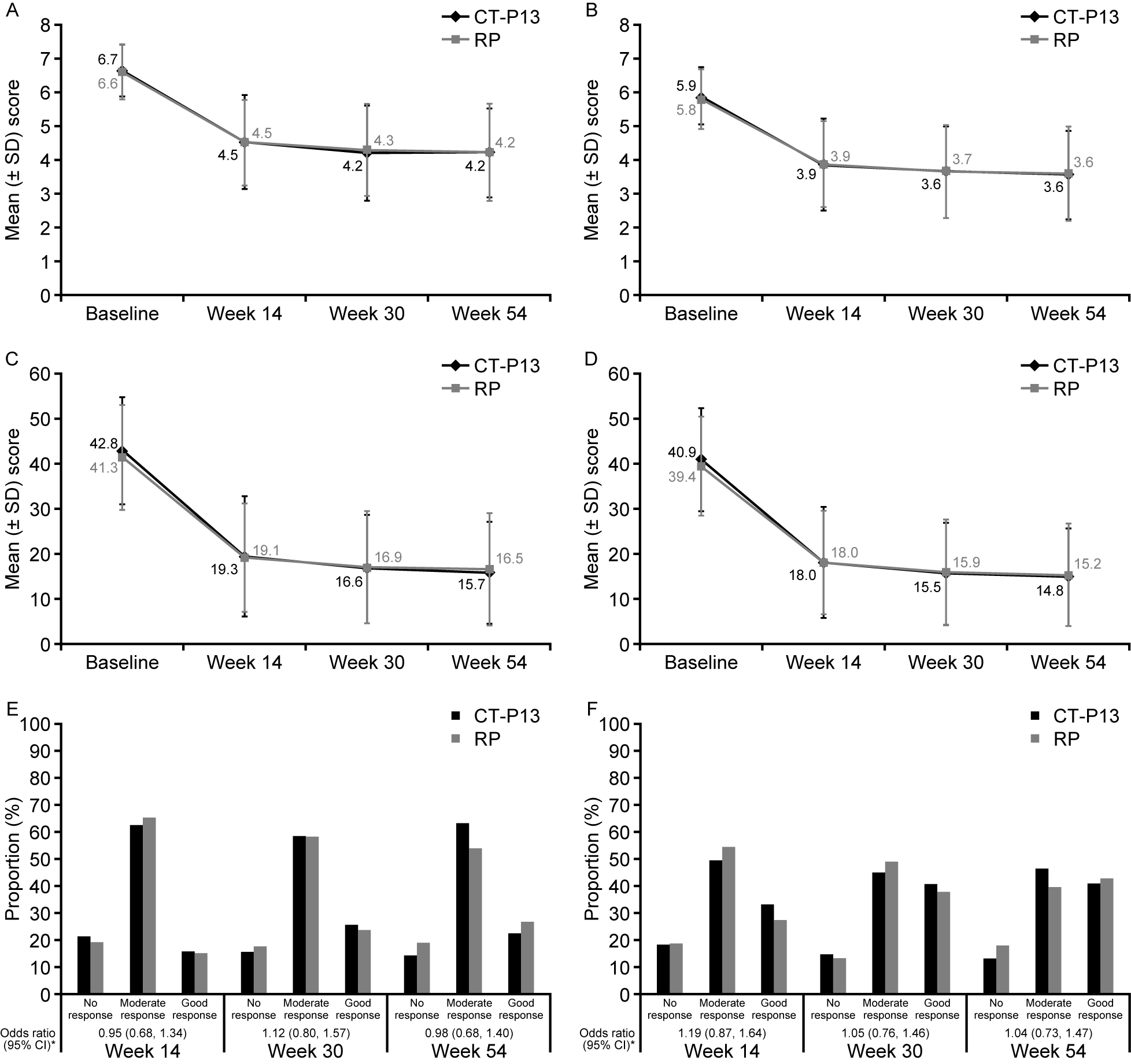

Supplement: Additional file 2: — Changes in efficacy parameters over time with CT-P13 and RP in the intent-to-treat population. (A) Disease activity based on DAS28-ESR; (B) Disease activity based on DAS28-CRP; (C) Disease activity based on SDAI; (D) Disease activity based on CDAI; (E) EULAR response criteria based on DAS28-ESR score; (F) EULAR response criteria based on DAS28-CRP score. *Proportional odds model with EULAR as response, treatment as a fixed effect, and region and CRP category as covariates. An odds ratio >1 implied that a patient who received CT-P13 had a higher likelihood of EULAR response than a patient who received RP. The proportional odds assumption implied that the relationship between each pair of outcome response was the same. CDAI, Clinical Disease Activity Index; CI, confidence interval; CRP, C-reactive protein; DAS28, Disease Activity Score in 28 joints; ESR, erythrocyte sedimentation rate; EULAR, European League Against Rheumatism; RP, reference product (i.e., reference infliximab); SD, standard deviation; SDAI, Simplified Disease Activity Index. (TIF 1420 kb) [file 13075_2016_981_MOESM2_ESM.tif]
