# Supplementary material for: A phase III randomized study to evaluate the efficacy and safety of CT-P13 compared with reference infliximab in patients with active rheumatoid arthritis: 54-week results from the PLANETRA study
Source: Arthritis Res Ther. 2016 Apr 2;18:82. doi: 10.1186/s13075-016-0981-6 (PMC4818886; doi:10.1186/s13075-016-0981-6)
Supplement: Additional file 3: — Improvement in patient-reported outcomes with CT-P13 and RP in the intent-to-treat population. (DOC 52 kb) [file 13075_2016_981_MOESM3_ESM.doc]

Additional File 3. Improvement in patient-reported outcomes with CT-P13 and RP in the intent-to-treat population.

|  | **CT-P13 (3 mg/kg)** | | | **RP (3 mg/kg)** | | |
| --- | --- | --- | --- | --- | --- | --- |
| **Time point** | **n** | **Actual result (mean ± SD)** | **Change from baseline (mean ± SD)** | **n** | **Actual result (mean ± SD)** | **Change from baseline (mean ± SD)** |
| **VAS score for the patient assessment of pain** | | | | | | |
| Baseline | 300 | 65.9 ± 17.5 | – | 302 | 65.5 ± 17.2 | – |
| Week 14 | 278 | 37.3 ± 22.7 | -28.5 ± 23.9 | 281 | 38.5 ± 22.6 | -27.1 ± 23.5 |
| Week 30 | 256 | 36.5 ± 23.1 | -29.3 ± 25.8 | 260 | 37.9 ± 24.0 | -27.7 ± 25.2 |
| Week 54 | 235 | 34.8 ± 21.2 | -30.6 ± 23.9 | 226 | 37.1 ± 24.7 | -28.7 ± 26.9 |
| **VAS score for the patient global assessment of disease activity** | | | | | | |
| Baseline | 300 | 65.7 ± 17.2 | – | 302 | 65.4 ± 17.0 | – |
| Week 14 | 278 | 36.7 ± 22.4 | -28.7 ± 23.2 | 280 | 39.9 ± 22.8 | -25.7 ± 24.7 |
| Week 30 | 255 | 37.4 ± 22.6 | -27.7 ± 26.3 | 260 | 38.5 ± 23.7 | -26.8 ± 26.0 |
| Week 54 | 234 | 34.7 ± 20.7 | -30.6 ± 24.4 | 226 | 38.5 ± 25.3 | -26.8 ± 27.8 |
| **HAQ estimate of physical ability** | | | | | | |
| Baseline | 300 | 1.61 ± 0.55 | – | 302 | 1.56 ± 0.59 | – |
| Week 14 | 278 | 1.05 ± 0.63 | -0.56 ± 0.56 | 282 | 1.04 ± 0.65 | -0.50 ± 0.52 |
| Week 30 | 256 | 1.01 ± 0.64 | -0.60 ± 0.59 | 261 | 1.02 ± 0.65 | -0.51 ± 0.57 |
| Week 54 | 235 | 0.98 ± 0.60 | -0.61 ± 0.61 | 226 | 1.01 ± 0.65 | -0.53 ± 0.60 |
| **SF-36 score (physical component summary)** | | | | | | |
| Baseline | 299 | 31.2 ± 6.1 | – | 302 | 31.8 ± 7.1 | – |
| Week 14 | 277 | 38.5 ± 7.7 | 7.3 ± 7.1 | 282 | 37.7 ± 7.9 | 5.7 ± 7.2 |
| Week 30 | 256 | 38.6 ± 7.9 | 7.2 ± 7.9 | 260 | 38.5 ± 8.1 | 6.7 ± 7.8 |
| Week 54 | 235 | 39.3 ± 7.5 | 7.7 ± 8.1 | 226 | 38.7 ± 8.8 | 6.9 ± 8.6 |
| **SF-36 score (mental component summary)** | | | | | | |
| Baseline | 299 | 36.9 ± 10.6 | – | 302 | 37.9 ± 11.1 | – |
| Week 14 | 277 | 43.2 ± 10.9 | 6.2 ± 10.2 | 282 | 44.3 ± 9.8 | 6.5 ± 10.4 |
| Week 30 | 256 | 43.9 ± 10.1 | 7.0 ± 10.0 | 261 | 45.0 ± 10.5 | 6.7 ± 10.6 |
| Week 54 | 235 | 44.0 ± 9.8 | 7.0 ± 10.4 | 226 | 45.2 ± 10.1 | 7.1 ± 11.4 |

HAQ, health assessment questionnaire; RP, reference product (i.e. reference infliximab); SD, standard deviation; SF-36, Medical Outcomes Study Short-Form Health Survey; VAS, visual analogue scale (mm).
