# Supplementary material for: A phase III randomized study to evaluate the efficacy and safety of CT-P13 compared with reference infliximab in patients with active rheumatoid arthritis: 54-week results from the PLANETRA study
Source: Arthritis Res Ther. 2016 Apr 2;18:82. doi: 10.1186/s13075-016-0981-6 (PMC4818886; doi:10.1186/s13075-016-0981-6)
Supplement: Additional file 4: — Overview of treatment-related serious adverse events occurring by severity (n [%]). (DOC 49 kb) [file 13075_2016_981_MOESM4_ESM.doc]

Additional file 4: Overview of treatment-related SAEs occurring by severity, n (%).

|  | **CT-P13**  **3 mg/kg (N=302)** | | | **RP**  **3 mg/kg (N=300)** | | |
| --- | --- | --- | --- | --- | --- | --- |
| **n (%)** | **Mild** | **Moderate** | **Severe** | **Mild** | **Moderate** | **Severe** |
| Lower respiratory tract infection | 1 (0.3) | 3 (1.0) | – | – | – | – |
| TB | – | 2 (0.7) | 1 (0.3) | – | – | – |
| Joint infection | – | – | 1 (0.3) | – | – | 1 (0.3) |
| Appendicitis | – | – | – | – | – | 1 (0.3) |
| Herpes virus infection | – | – | – | – | – | 1 (0.3) |
| Sepsis | – | – | – | – | – | 1 (0.3) |
| Wound infection | – | 1 (0.3) | – | – | – | – |
| Infusion-related reaction | – | 2 (0.7) | 4 (1.3) | 2 (0.7) | 1 (0.3) | 1 (0.3) |
| Blood glucose increased | – | – | – | – | 1 (0.3) | – |
| Neutropenia | – | – | 1 (0.3) | – | – | – |
| Musculoskeletal chest pain | – | – | 1 (0.3) | – | – | – |
| Flare in RA activity | – | – | 1 (0.3) | – | – | – |
| Breast cancer | – | – | – | – | – | 1 (0.3) |
| Ovarian cancer | – | – | – | – | – | 1 (0.3) |
| Renal neoplasm | 1 (0.3) | – | – | – | – | – |
| Endometrial hyperplasia | – | 1 (0.3) | – | – | – | – |
| Uterine hemorrhage | – | – | – | – | 1 (0.3) | – |
| Cerebrovascular disorder | – | – | 1 (0.3) | – | – | – |
| Acute coronary syndrome | – | – | – | – | – | 1 (0.3) |
| Thrombophlebitis | – | – | 1 (0.3) | – | – | – |
| Tympanic membrane perforation | – | – | 1 (0.3) | – | – | – |
| Pyrexia | – | – | – | 1 (0.3) | – | – |

RA, rheumatoid arthritis; RP, reference product (i.e. reference infliximab); SAE, serious adverse event; TB, tuberculosis.

Note: The event was considered to be related if the relationship was defined as “possible,” “probable” or “definite”.
